# Bolder guppies do not have more mating partners, yet sire more offspring

**DOI:** 10.1186/s12862-019-1539-4

**Published:** 2019-11-14

**Authors:** Magdalena Herdegen-Radwan

**Affiliations:** Department of Behavioural Ecology, Faculty of Biology of Adam Mickiewicz University, Uniwersytetu Poznańskiego 6, 61-614 Poznan, Poland

**Keywords:** Personality, Reproductive success, Female preferences, Fitness

## Abstract

**Background:**

Intra-individual stable but inter-individually variable behaviours, i.e. personalities, are commonly reported across diverse animal groups, yet the reasons for their maintenance remain controversial. Therefore, studying fitness consequences of personality traits is necessary to discriminate between alternative explanations.

**Results:**

Here, I measured boldness, a highly repeatable personality trait, and reproductive success in male guppies, *Poecilia reticulata*. I found that bolder males had higher reproductive success than their shyer conspecifics and they sired offspring with females who had larger clutches.

**Conclusions:**

This result provides direct evidence for fitness consequences of boldness in the guppy. It suggests that the effect may be driven by bolder males mating with more fecund females.

## Background

In the last few decades, the existence of consistent behaviour patterns that differ among individuals, termed personality traits, has been described in many species [1–3]. An early hypothesis regarding the evolution of such traits suggested that they evolve neutrally, by the sole mechanisms of the mutation-drift balance [4]. An alternative explanation was that personality traits could influence an individual’s fitness, and in the last few decades researchers have indeed found some evidence for associations between personality traits and fitness components (reviewed in [5]). Both, studies supporting such associations [6–8], as well as not [9–11], have been published to date, and thus no consistent pattern of the general effect of personalities on fitness has yet emerged.

Boldness, defined as the willingness to be active in a situation when such behaviour is potentially risky (e.g. [12, 13]), is one of the most studied personality traits across animal species. In the existing literature, some studies have found support for a link between boldness and reproductive success, a pivotal component of individual fitness. In a meta-analysis from 2008, Smith and Blumstein [6] reported a moderately positive effect of boldness on reproductive success, but only in captive animals. Several studies have been published since, of which some have reported a similar relationship, for example, higher reproductive success of bolder individuals in Eastern chipmunks, *Tamias striatus* [14], and zebrafish, *Danio rerio* [15, 16], and longer survival of bolder guppies when exposed to a predator [17]. In contrast, another study found a negative association between boldness and female fecundity in mosquitofish (*Gambusia affinis*) [18], indicating that negative consequences of being bold exist in this species, it is however important to note that lower fecundity may not necessarily translate into reduced reproductive success. Similarly, some studies suggest a negative effect of boldness on survival (reviewed in [6]). In addition to these positive and negative associations a number of studies found no association between boldness and fitness (e.g. [9, 10]). Thus, to date we have no overall consensus regarding the relationship between boldness and fitness. More data are needed to make generalizations about non-neutrality, and direction of the effect.

Here, I used guppies to examine the association between boldness and male reproductive success. The guppy is a small tropical freshwater fish which has served as a model in studies of evolution and sexual selection (e.g. [19–22]). In the last three decades it has also served increasingly as a model for studying the causes and consequences of personality variation (e.g. [23–27]). Among important determinants of guppy male reproductive success, carotenoid colouration stands out as the ornament most consistently preferred by females across populations (e.g. [21, 23, 28]), and is often correlated to the number of offspring sired [29]. However, Godin and Dugatkin [23] showed that females who previously found colourful males more attractive, switched to choosing males based primarily on their boldness when given the opportunity to assess both traits of potential partners simultaneously. Male boldness was manipulated by placing each male in a transparent tube at different distances from a predator model, so that females were presented with a full range of possible combinations of colour and perceived boldness of their potential partners. Males perceived as bolder (i.e. closer to a predator) were consistently preferred by the experimental females, independent of their carotenoid colouration.

Here, I tested if boldness is associated with reproductive success for guppy males. Eighty randomly chosen males were scored for boldness in an emergence test, and then allowed to mate with groups of females. Male reproductive success, measured as the number of offspring he sired with all mating partners, assessed with parentage analysis based on a set of microsatellite markers, was then correlated with his boldness level. Additionally, to account for the confounding effect of ornamental traits and to test if they are associated to personality in my study population, I measured male carotenoid colouration, i.e. the relative area of their carotenoid spots.

## Results

Of the 70 males for which complete data on boldness and colouration was collected, 30 (43%) successfully sired offspring, and the mean number of offspring sired by a father was 5.9. The distribution of boldness scores across aquaria is represented in Additional file 1: Figure S1. The clutches of 18 females were sired by a single male, 16 females had clutches which paternity was assigned to two males, and two females had clutches assigned to three males.

**Fig. 1:**
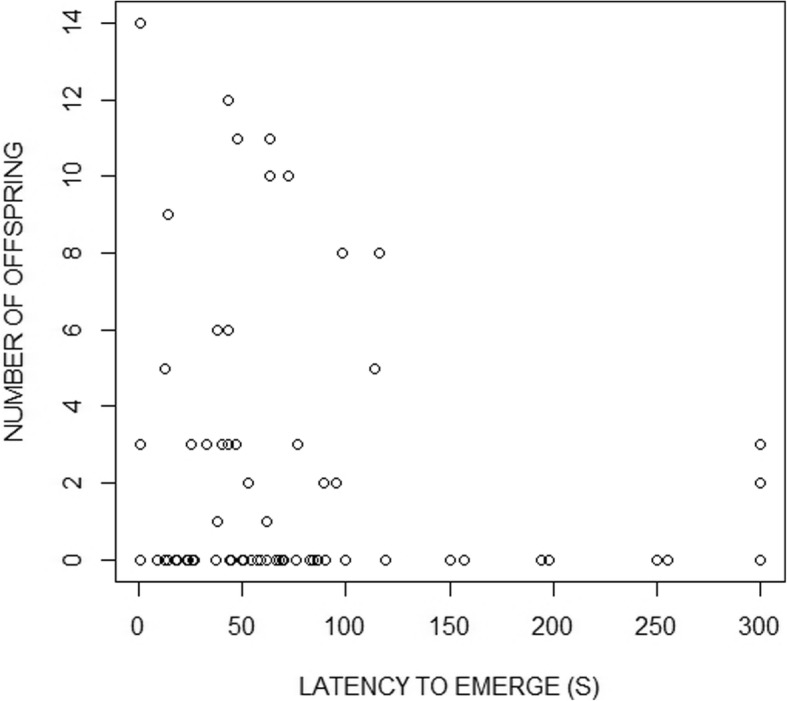
The relationship between a males’ boldness, measured as latency (in seconds) to emerge from the shelter, and his reproductive success, measured as the number of offspring sired

Boldness, orange area and body size did not predict success or failure of a male to reproduce (boldness: z_7,69_ = − 0.82, *p* = 0.41; orange area: z_7,69_ = 0.76, *p* = 0.45; body size: z_7,69_ = − 0.01, *p* = 0.99). There was a significant effect of boldness on the number of offspring sired: bolder males sired more offspring (z_7,71_ = − 0.38, *p* = 0.011; Fig. 1). Colouration and body size did not affect male reproductive success (Table 1; Additional file 2: Figure S2, Additional file 3: Figure S3, respectively). Apart from boldness, the number of a males’ mating partners had a strong effect on male reproductive success (z_7,71_ = 0.89, *p* < 0.000). However, boldness did not significantly explain male number of mates (z_7,71_ = − 1.33, *p* = 0.183; Additional file 4: Figure S4, full model in Table 2), which implies that the positive effect of boldness on male reproductive success was not mediated by bolder males mating with a larger number of females.

**Table 1:** Predictors of male reproductive success, i.e. number of offspring sired. Generalized linear mixed model with a zero-inflated distribution of model residuals was used. Significant *p* values are denoted with asterisk

Variable	Estimate	S.E.	z	*P*
Boldness	−0.310	0.149	−2.557	0.011*
N of partners	0.893	0.157	5.671	> 0.000*
Orange area	−0.108	0.131	−0.820	0.412
Body size	0.213	0.144	1.477	0.140

**Table 2:** Predictors of the number of male mating partners, i.e. females who gave birth to those males’ offspring. Generalized linear mixed model with a zero-inflated distribution of model residuals was used

Variable	Estimate	S.E.	z	*P*
Boldness	−0.249	0.217	0.593	0.553
Orange area	0.096	0.177	0.543	0.587
Body size	−0.147	0.222	−0.659	0.510

**Fig. 2:**
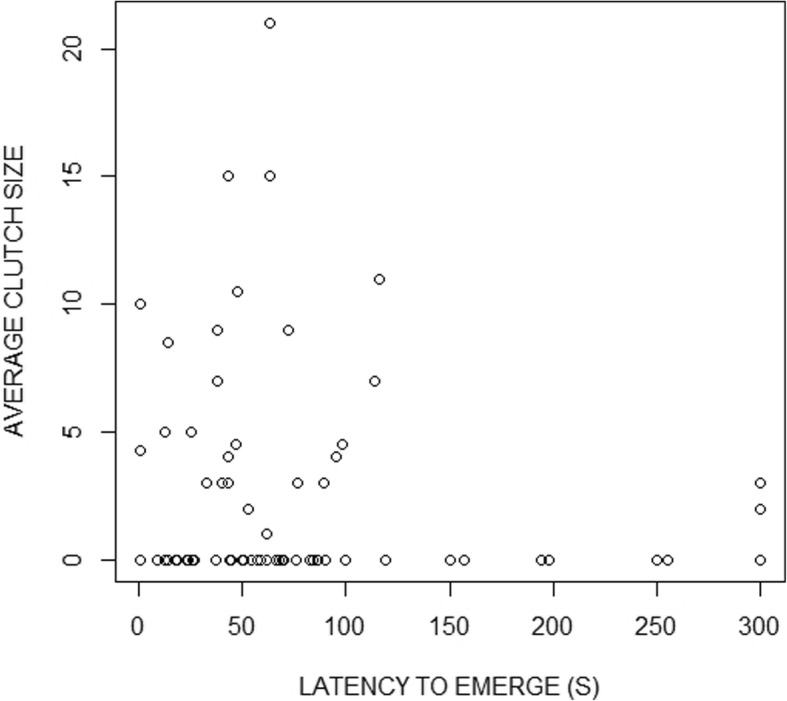
The relationship between a males’ boldness, measured as latency (in seconds) to emerge from the shelter, and average clutch size of the females that he mated with

To further explore the mechanism of potential effect of boldness on reproductive success, I tested if bolder males were mated to females with significantly larger clutches. I found a positive association of a males' boldness with the average clutch size across all females he sired offspring with (z_7,71_ = − 2.84, *p* = 0.005; Fig. 2).

## Discussion

Although animal personalities have been extensively studied for over 30 years, we still have limited knowledge about the fitness consequences of personality traits [6, 30]. Here, I looked for associations between boldness, one of the most-studied personality traits in animals, and reproductive success, the crucial component of individual fitness, in a fish species. I found a positive and significant effect of guppy male boldness, measured by their propensity to start exploring a new and possibly dangerous environment, on their reproductive success, measured as the number of offspring sired. This result is consistent with a study on zebrafish, which reported a positive association between male boldness and the number of offspring sired [15].

The higher reproductive success of bold guppy males found in the present study could result from female choice, as earlier reported by Godin and Dugatkin [23], who demonstrated female preference for bolder males. However, this study did not provide direct evidence for the importance of boldness for guppy male fitness, as the authors did not allow for matings. Here, I show that bolder males indeed have higher reproductive success than their shyer male conspecifics. Although the number of mating partners also had a strong effect on male reproductive success, as could be expected if bold males were preferred as mating partners [23], boldness was however not associated with the number of females a male successfully sired offspring with. In line with this result, I also found no effect of boldness on the probability of success or failure to reproduce. This suggests, that some other factor/s rather than increased mating success resulting from sexual attractiveness, is responsible for the effect of boldness on reproductive success.

One possibility could be that the sperm of bold males outcompetes that of shy males. Indeed, Gasparini et al. [31] reported an association between boldness and the number of sperm in the guppy, while Evans et al. [32] showed that intrinsic sperm quality of guppy males plays a crucial role in sperm competition after fertilisation, and the key feature conferring higher success is sperm velocity [33]. Alternatively, the determining factor could be female cryptic post-mating preferences, which have been recently documented in the guppy [34, 35]. However, an intriguing result of this study, i.e. the association between successful male boldness and the average clutch size of his mating partners, suggests another possibility. Since bigger guppy females produce more offspring [36, 37], this result may suggest assortative mating among bolder males and larger females. Indeed, male preference for larger more fecund females has been previously found in this species [38, 39]. Thus, if bolder males are better at gaining access to the most preferred females (either through competition or choice), they are expected to successfully mate with large females. Unfortunately, female size was not measured in the current study. Most variation in size between guppy females stems from female age (they grow throughout their lives). Although females used in the current study were of similar age (5 to 9 months), some size variation between females, resulting in clutch size variation, cannot be excluded. A future experiment is needed to check if bolder males mate preferentially with larger females. Alternatively, bolder males may confer some fitness benefits to their offspring, for example, better survival to parturition. A similar effect has been previously reported in zebra fish, where eggs sired by bold males had improved viability [15]. This could be the case here, as I measured male reproductive success as the number of offspring after birth. Thus, “bigger clutch” ascribed to a female may be an effect of better juvenile survival, rather than of bigger clutch produced. Discrimination between the alternatives discussed above will require further research.

Irrespective of underlying mechanism, my data show that male boldness is associated with higher fitness. This highlights the need for an explanation for the presence of variation in personalities. The experiment here was conducted in the absence of predators. Under predation pressure there could potentially be a boldness-related trade-off between individual whole-life reproductive success and survival, since bold fish behave in a more risky way and are thus expected to be under increased risk of predation. Such scenario seems plausible, and indeed, a study by Dugatkin [40] found a negative effect of guppy boldness on survival. Smith and Blumstein [17] however, showed when exposed to a predator there was a positive effect of boldness and exploration on survival in this species. Furthermore, two studies [41, 42] found differences among guppy populations inhabiting high and low predation sites, but counter intuitively they observed higher levels of boldness under stronger predation pressure. Thus, further exploration of the possible trade-offs between different components of fitness as drivers of variation in guppy personality traits is needed.

I found no effect of male orange colouration on the number of offspring fathered. Carotenoid colouration is known to be costly to express and to be an honest indicator of quality in many species [43]. The trait measured in this study was the relative area of carotenoid colouration. Previous studies on guppies, based on the same measure of colouration, have shown higher reproductive success of colourful males [29], cryptic female choice for colourful males [44], and that colourful males produce faster and more viable sperm, which should increase their reproductive success [45]. However, other authors have reported no effect of colouration on guppy sperm competitiveness [46], no preference of females of a related Poecilia species (*P.picta*) for colourful males [47], and no fecundity benefits for guppy females mating with more colourful males [48], which is in line with a study on zebrafish [16]. This indicates that factors that we still do not understand contribute to the overall effect of colouration on male guppy reproductive success.

## Conclusions

The present study provides direct evidence for fitness consequences of boldness in the guppy. Bolder guppy males were found to have higher reproductive success. This effect was not directly driven by higher number of females they sired offspring with. It may be due to bolder males mating more often with females who gave birth to more offspring, or by positive association between male boldness and offspring survival. These possibilities highlight some potential avenues for future research to address. It could also be interesting to investigate the fitness consequences of boldness in the next generation by keeping track of F1 and comparing life history traits (e.g. survival to reproduction, attractiveness or mating success) of offspring sired by bold and shy males.

## Methods

### Study population

Experimental fish were descendants of wild-caught Trinidadian guppies collected from Tacarigua. Fish in stock and throughout the experiment were kept in stable conditions: temperature 25 ± 1 °C; 12:12 h light/dark regime; and fed twice per day, once with commercial dry flakes and once with nauplii of *Artemia* sp.

### Experimental design

For logistical reasons the experiment was conducted in two blocks. The first block consisted of 20 males and the second comprised 60. Males in each block were randomly caught from the stock population and put into 3 L tanks in a ZebTEC machine (Tecniplast), which allows to keep identical water conditions in all experimental tanks. After spending 3 days in these conditions, their boldness was measured.

For the emergence test, an aquarium (40x20x30cm) filled to a depth of 10 cm of water was used. The aquarium walls were covered from outside with opaque plastic sheets, to avoid fish being distracted. The aquarium contained a dark, plastic box (10x10x10 cm) which served as refuge and was placed close to one of the aquarium walls. At the beginning of the trial a male was put into the refuge box through a hole in the ceiling, which was immediately covered. After 5 min of acclimatisation the door in the front wall of the box was removed, which was done discretely (by pulling the door up by a string at the side of the aquarium) without the experimenter being seen by the fish. Boldness was measured as the time taken by the male to emerge from the refuge box (i.e. when his whole body was visible through the camera suspended above the aquarium). Males who emerged earlier into the open space of the unfamiliar aquarium were considered bolder. A maximum score of 300 s was assigned to those fish that did not come out within 5 min of removing the door. Immediately after the trial, the fish were released back to the home aquarium to avoid familiarization with the test arena.

Repeatability of emergence test is high within the population studied here (0.64, CI 0.60–0.68, see [49] and Table 2 therein). In short, 51 males were tested twice following the procedure described above, with a 1 week interval between the tests. Repeatability was calculated according to Lessells and Boag [50], by dividing the among-individual variance by the sum of the among- and within-individual variances.

After completing the trials, males were randomly and blindly with respect to their boldness score, assigned to one of two (in block 1) or six (in block 2) aquaria (40x30x30 cm), 10 males per aquarium, and allowed to mate for 1 week with mature (5 to 9 months old) virgin females from the stock population, also 10 per aquarium. Such an arrangement, as opposed to forming individual mating pairs, allowed for male-male competition, and gave females the opportunity to compare and mate with a number of different partners. Thus, this approach enabled me to minimise potential differences in mating motivation and investment in reproduction for both sexes. After 1 week of mating, males were removed, the tips of their tail fins were taken for DNA analysis, and they were photographed on their left side under anaesthesia (MS-222). Females were kept in breeding chambers until parturition, after which their tail-fin tips were also taken. Tail-fin tips were sampled from all male and female F1 guppies. All fin samples were stored in 95% ethanol until DNA extraction. All tests and measurements were carried out blindly with respect to the results of the other analyses.

Body area (excluding tail fin) and the area of carotenoid (orange, red, and yellow) spots of all males from the parental generation were measured from photographs using Image J software [51]. The relative area of carotenoid spots was measured as the sum of the area of all spots divided by the body area.

### Molecular analyses

DNA was extracted from tail-fin samples using the MagJet Genomic DNA Kit (Thermo Fisher Scientific) according to the manufacturer’s guidelines. In order to assign F1 individuals to their parents, all individuals were screened for variation at six previously described microsatellite loci: Pret-27 [52], G183 [53], TACA033, AG11 [54], G75 [53], and Pret77 [52]. DNA was amplified in two multiplex polymerase chain reactions using PCR Master Mix (Qiagen); one reaction amplified the first three loci while the other amplified the last three. One primer of each primer pair was fluorescently labelled to enable its identification. The 10-uL PCR mixture contained 5 uL of Master Mix, 0.2–0.4 uM of each primer, and 20–100 ng of genomic DNA. The reaction conditions were as follows: a 15-min denaturation step at 95 °C, followed by 36 cycles of 30 s at 94 °C, 1 min at 52 °C, and 1 min at 72 °C, then 10 min of final extension at 72 °C. PCR products were mixed with a GeneScan LIZ500 size standard and electrophoresed on an ABI 3130xl Genetic Analyser. Genotyping was performed using the ABI software GeneMapper 4.0.

### Statistical analysis

Parentage was assigned using COLONY 2.0 [55]. Each of the eight groups of 10 males, 10 females, and their offspring was analysed separately, using the full-likelihood method. In each case, one long run was performed, with the following parameters: high likelihood precision, polygamy allowed for both sexes, and no sibship prior. A father or mother were considered parents of an individual if the associated probability of assignment of the putative offspring was above 0.8 (in 97% of cases this value was above 0.9). The number of offspring assigned as sired by a male was the measure of male reproductive success.

A generalised linear mixed model (GLMM) with a binomial distribution of model residuals was used to test for the effect of personality on the probability that a male reproduces. Another GLMM, with a zero-inflated distribution of model residuals, was used to test for the effect of personality on the reproductive success. All males from parental generation were included in those analyses. Both models included boldness, male orange area, male body size, and aquarium (random factor). In the second model, also the number of mating partners was incorporated as fixed factor. Throughout the paper, ‘mating partner’ is used to refer to any female which a male successfully sired offspring with. Block was not entered in the analyses, as its associated variance was accounted for by a random factor aquarium.

The effect of boldness on the number of partners was analysed with a separate GLMM with a zero-inflated distribution of model residuals. In this model also male orange area and body size were included as covariates, and aquarium as random factor.

To explore if a males' boldness is associated with clutch size of his mating partners, I run a GLMM with a zero-inflated distribution of model residuals with average number of young produced by females mated with a given male as response variable and boldness as a predictor. All continuous variables in all analyses were z-scaled. All tests were performed in R v. 3.6.0 [56], in the packages lme4 [57] and glmmTMB [58].

## Supplementary information

**Additional file 1. Figure S1.** Distribution of male boldness scores across aquaria (1–8) and blocks (A, B). The boxes represent median ± interquartile range, whiskers denote min and max values, outliers are marked with open dots.

**Additional file 2: Figure S2.** The relationship between a males’ reproductive success, measured as the number of offspring sired, and his colouration, measured as the relative area of orange spots.

**Additional file 3: Figure S3.** The relationship between a males’ reproductive success, measured as the number of offspring sired, and his body size measure, i.e. body area excluding fins.

**Additional file 4: Figure S4.** The relationship between a males’ boldness, measured as latency (in seconds) to emerge from the shelter, and the number of females he sired offspring with.

**Additional file 5:** Raw data generated during this study, including each male ID, the aquarium and block he was assigned to, boldness score, number of offspring sired, number of mating partners, orange spots area, body size (excluding fins) and mean clutch size among all of his mating partners.

## Data Availability

The raw data generated during this study are included in Additional file 5.

## Abbreviations

GLMM: Generalised linear mixed model
MS-222: Tricaine methanesulfonate
