# Supplementary figures and images for: Bolder guppies do not have more mating partners, yet sire more offspring
Source: BMC Evol Biol. 2019 Nov 14;19:211. doi: 10.1186/s12862-019-1539-4 (PMC6857137; doi:10.1186/s12862-019-1539-4)

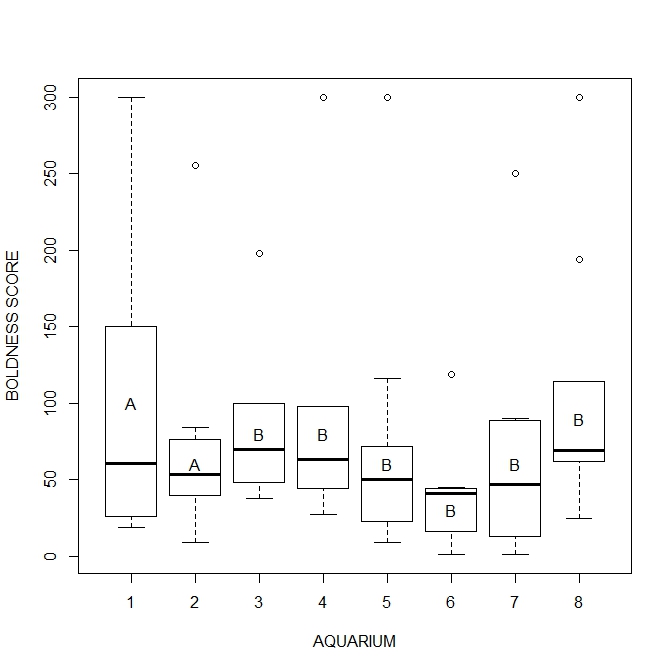

Supplement: Supplementary file 1 — Additional file 1. Figure S1. Distribution of male boldness scores across aquaria (1–8) and blocks (A, B). The boxes represent median ± interquartile range, whiskers denote min and max values, outliers are marked with open dots. [file 12862_2019_1539_MOESM1_ESM.jpeg]

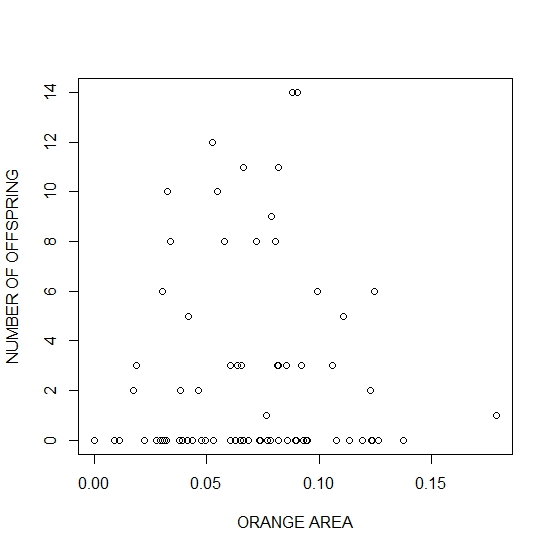

Supplement: Supplementary file 2 — Additional file 2: Figure S2. The relationship between a males’ reproductive success, measured as the number of offspring sired, and his colouration, measured as the relative area of orange spots. [file 12862_2019_1539_MOESM2_ESM.jpeg]

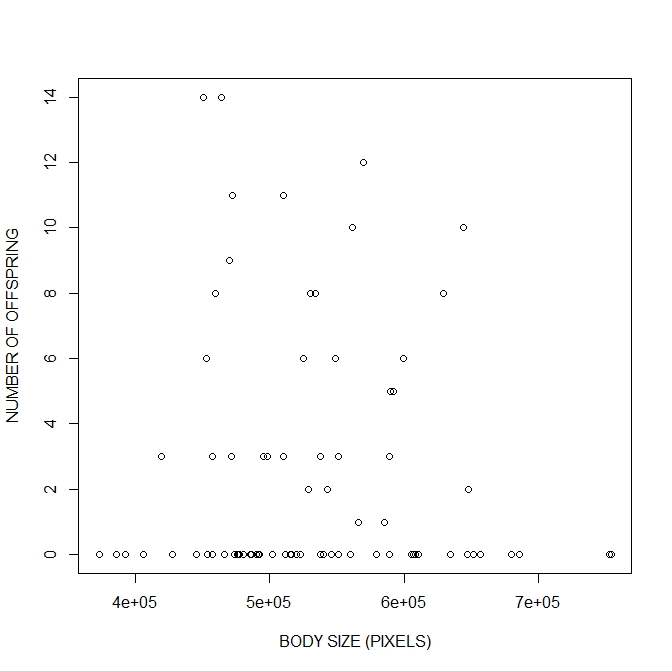

Supplement: Supplementary file 3 — Additional file 3: Figure S3. The relationship between a males’ reproductive success, measured as the number of offspring sired, and his body size measure, i.e. body area excluding fins. [file 12862_2019_1539_MOESM3_ESM.jpeg]

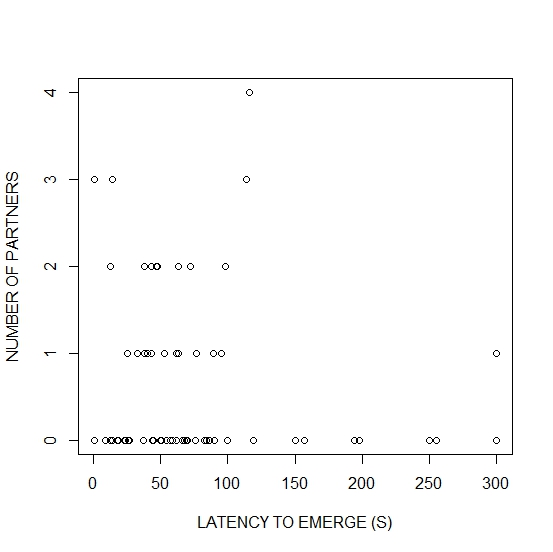

Supplement: Supplementary file 4 — Additional file 4: Figure S4. The relationship between a males’ boldness, measured as latency (in seconds) to emerge from the shelter, and the number of females he sired offspring with. [file 12862_2019_1539_MOESM4_ESM.jpeg]
